# Accounting for confounding by time, early intervention adoption, and time-varying effect modification in the design and analysis of stepped-wedge designs: Application to a proposed study design to reduce opioid-related mortality

**DOI:** 10.21203/rs.3.rs-103992/v1

**Published:** 2020-11-12

**Authors:** Lior Rennert, Moonseong Heo, Alain H. Litwin, Victor De Gruttola

**Affiliations:** a Department of Public Health Sciences, Clemson University, Clemson, U.S.A.; b University of South Carolina School of Medicine, Greenville, SC, USA; c Prisma Health, Department of Medicine, Greenville, SC, USA; d Clemson University School of Health Research, Clemson, SC, USA; e Department of Biostatistics, Harvard T.H. Chan School of Public Health, Harvard University, Boston, U.S.A.

**Keywords:** Stepped wedge design, Cluster randomized trials, Study design, Confounding, Secular trends, Analysis, Opioids, COVID-19, Epidemic, Pandemic

## Abstract

**Background:**

Stepped-wedge designs (SWDs) are currently being used in the investigation of interventions to reduce opioid-related deaths in communities located in several states. However, these interventions are competing with external factors such as newly initiated public policies limiting opioid prescriptions, media awareness campaigns, and COVID-19 social distancing mandates. Furthermore, control communities may prematurely adopt components of the intervention as they become available. The presence of time-varying external factors that impact study outcomes is a well-known limitation of SWDs; common approaches to adjusting for them make use of a mixed effects modeling framework. However, these models have several shortcomings when external factors differentially impact intervention and control clusters.

**Methods:**

We discuss limitations of commonly used mixed effects models in the context of proposed SWDs to investigate interventions intended to reduce opioid-related mortality, and propose extensions of these models to address these limitations. We conduct an extensive simulation study of anticipated data from SWD trials targeting the current opioid epidemic in order to examine the performance of these models in the presence of external factors. We consider confounding by time, premature adoption of components of the intervention, and time-varying effect modification— in which external factors differentially impact intervention and control clusters.

**Results:**

In the presence of confounding by time, commonly used mixed effects models yield unbiased intervention effect estimates, but can have inflated Type 1 error and result in under coverage of confidence intervals. These models yield biased intervention effect estimates when premature intervention adoption or effect modification are present. In such scenarios, models incorporating fixed intervention-by-time interactions with an unstructured covariance for intervention-by-cluster-by-time random effects result in unbiased intervention effect estimates, reach nominal confidence interval coverage, and preserve Type 1 error.

**Conclusions:**

Mixed effects models can adjust for different combinations of external factors through correct specification of fixed and random time effects; misspecification can result in bias of the intervention effect estimate, under coverage of confidence intervals, and Type 1 error inflation. Since model choice has considerable impact on validity of results and study power, careful consideration must be given to choosing appropriate models that account for potential external factors.

## 1. Introduction

**Figure 1.**
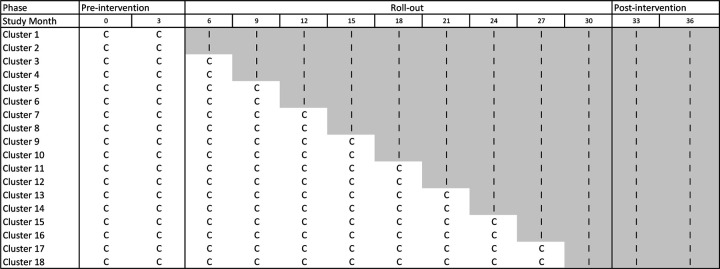
Proposed SWD for 18 South Carolina communities. ‘C’ indicates cluster receives control and ‘I’ indicates cluster receives intervention. All clusters are in the control condition during the pre-intervention phase (months 0 through 6). During the roll-out phase (months 6 through 33), two clusters crossover to the intervention condition at the beginning of each time period. In the follow-up phase (months 33–39), all clusters receive the intervention.

Stepped wedge designs (SWD) are a uni-directional crossover design in which clusters switch from the control to the intervention condition at varying time points. The first phase is usually a baseline period in which no clusters receive intervention. During the second phase, clusters are randomly assigned to intervention at pre-selected time points until all clusters receive the intervention. The third phase corresponds to the follow-up period in which all clusters receive the intervention. An example of a SWD is provided in Figure 1 and discussed in Section 1.1.

SWDs can be useful in public health settings where rolling out the intervention to all clusters at once is infeasible; they also ensure that all clusters in the study eventually receive the intervention.^1, 2^ The SWD is particularly suitable for implementing and evaluating complex health interventions.^3–5^ The current COVID-19 pandemic and opioid epidemic provide settings in which such designs may prove useful; effective combinations of interventions are needed as soon as possible, but rolling them out to each community in a short time period may not be feasible. In this paper, we will focus on recently proposed SWDs to combat the opioid epidemic. In 2019, the National Institute on Drug Abuse (NIDA) awarded Kentucky and Ohio roughly $100 million each to implement an integrated set of interventions in high-risk communities using a SWD, with the objective of reducing opioid overdose deaths by 40% over a three-year period.^6–9^

Like most clinical trial designs, SWDs may be impacted by external factors that influence the primary outcomes. The term ”rising tide” has been used to describe the situation when there is a drift towards improvement in the outcomes due to factors external to the study.^10^ A rising tide may be seen in the current opioid epidemic, where the severity of this crisis has led to new public policies, media awareness campaigns, and external interventions that will improve outcomes in concurrence with any proposed interventions. For example, public policies were implemented to limit opioid prescriptions in both Kentucky and Ohio in the summer of 2017.^11^ In addition, the Center for Disease Control and Prevention launched intensive media awareness campaigns on the dangers of opioids in these states in September of 2017.^12^ In the current COVID-19 pandemic, interventions rolled out at the community level will compete with social distancing measures, novel treatments, and other interventions aimed at improving patient care. Such external factors may confound estimates of the intervention effect estimate in SWDs. This occurs when (i) factors external to the study (e.g., new public health policy) influence the primary outcomes over time, and (ii) the proportion of communities exposed to the intervention also increases with calendar time. This situation has been referred to as confounding by calendar time;^2, 13^ an example of such confounding is the rising tide described above.

Failure to account for confounding by time might result in severely biased intervention effect estimates,^2, 3, 13–19^ and lead to both Type 1 and Type 2 errors. Hussey and Hughes suggested the use of mixed effects models for analyzing data from SWD.^16^ To account for potential confounding by time, they recommend the incorporation of fixed time effects. However, models that incorporate secular trends common to all clusters may not be appropriate in this setting, because only some of the communities may be exposed to the external factors. Furthermore, the impact of these external factors on the outcome will likely differ in each community.

For these reasons, Girling and Hemming, Hooper et al., and Hemming et al. have suggested incorporating random cluster-by-time effects into the mixed effects models.^13, 17, 20^ This class of models captures confounding by time through these random effects. This is especially useful when the timing and level of exposure to external factors are unknown. However, these models implicitly assume that the random effect variance is the same for all clusters across all time points, and that the random time effects are independent within each cluster. Kasza et al. propose models with more general within-cluster correlation structures.^21^ These models are more applicable to the scenarios discussed here, since outcomes within a community are more likely to be similar for time periods before or after exposure to external factors. Furthermore, since exposure to external factors may increase with time, variation in the outcome may change with time as well.

The models described above assume that the random effects are identically distributed across clusters. This assumption may not appropriate in settings where the impact of external factors systematically differs between intervention and control communities. Kasza et al showed that the random effect covariance structure can have an important impact on the sample size and power,^21^ and that misspecification of this covariance structure can lead to biased estimates of the intervention effect.^22^

Given the discussion above, we consider three mechanisms through which time-varying external factors can impact SWD studies: a) confounding by the time of the effects of interest, b) inducing or facilitating non-compliance, and c) time-varying effect modification. The models we discuss below can accommodate all of these mechanisms, but interpretation of results requires consideration of the way in which external factors are operating. Noncompliance might arise, for example, through exposure to state-wide policies and media awareness campaigns that cause control communities to adopt available components of the intervention prior to the scheduled roll-out time. Premature adoption of components of the intervention may be seen as a form of rising tide; but its impact in this case arises through the effect of time on exposure to intervention. This differs conceptually from confounding by time in which the outcome, measured over time, is impacted by confounding factors that are unrelated to the intervention itself. Differential impact of external factors on intervention compared to control communities may be viewed as time-varying effect modification; we return to this point in the discussions below.

This paper discusses the issues described above in the context of a proposed SWD to reduce opioid-related mortality in South Carolina, and shows these issues can be addressed through appropriate choice of mixed effects models, with regard to both fixed time effects and random effect covariance structure. Our objectives are to (1) consider the implications of assumptions regarding the impact of external factors for choice of mixed-models – with a focus on our motivating example; (2) extend these models to accommodate different combinations of external factors; (3) conduct an extensive simulation study to examine the performance of mixed effects models under different model assumptions. Performance is assessed based on the bias, confidence interval coverage, power, and Type 1 error of the intervention effect estimate. We describe more fully what this estimate represents below. An important component of our simulation study is the generation of latent external factors from different distributions than that assumed by the model, i.e. normal distribution for random effects. This allows for investigation of the robustness of our methods to violations of model assumptions.

The outline of this paper is as follows. Section 1.1 introduces the motivating example. In Section 2, we examine existing mixed effects models for SWD, discuss their limitations, and introduce alternative models that improve robustness to different combinations of external factors. Section 3 conducts an extensive simulation study to examine the adequacy of these mixed-effects models under different scenarios regarding external factors. Discussion, extensions, and concluding remarks are provided in Section 4.

### 1.1. Motivating example: HEALing Communities Study

The HEALing Communities Study: Developing and Testing an Integrated Approach to Address the Opioid Crisis^7^ is used as an example to illustrate confounding by time in SWD. The purpose of this initiative was to develop and integrate a set of evidence based interventions using cluster randomized trials to reduce opioid overdose fatalities by 40% over a 3-year period in states nationwide. In response to this research funding announcement, our research team proposed a SWD to implement a comprehensive external facilitation intervention in 18 of South Carolina’s counties that were hit hardest by the opioid crisis.

The proposed SWD design is displayed in Figure 1. Each cluster consists of all individuals in a given county. Each time interval corresponds to a 3-month study period. The pre-intervention phase consists of two time periods between (study) month 0 and month 6, in which all clusters are in the control condition. The roll-out phase consists of nine time periods between months 6 through 33, where two clusters are randomly assigned to receive the intervention at the beginning of each time period. The post-intervention phase, in which all clusters receive the intervention, occurs between months 33 through 39. Data pertaining to the communities considered in our proposal are provided in Supplementary Table 1. The outcome, recorded at the end of each time period, consists of the total number of opioid overdose deaths in each cluster during the 3-month time interval.

The proposed external facilitation intervention included the following components: 1) Integration of screening, intervention, and referral to treatment within health care settings; 2) implementation of programs and providers prescribing medication-assisted treatment and linkage to such treatment for people with opioid use disorder (OUD); 3) implementation of evidence-based school and community-based OUD prevention programs; and 4) increasing availability and use of naloxone by first responders and the community. Given the amount of effort and resources currently directed to the fight against the opioid epidemic, there is potential for other events to affect the outcome. For example, in 2018 Executive Order No. 2017–43 was passed in South Carolina; this order set a 5-day limit for certain opioid prescriptions.^23^ Also in 2018, the South Carolina Division of Alcohol and Other Drug Abuse Services rolled out a media campaign to raise community awareness of opioid addiction.^24^ This campaign included digital, social, and traditional media tactics, and was intended to cover all counties in South Carolina. These external factors are part of a rising tide of interventions and are expected to reduce the opioid-related death count over time. Failing to account for this rising tide in an analysis will cause an upward bias in the estimation of the intervention effect. Similarly, an influx of synthetic opioids into the population will likely be associated with an increase in the death rate over time. A failure to account for this will cause underestimation of the intervention effect and may lead to Type II error. Another possibility is that control communities prematurely adopt components of the intervention before the scheduled roll-out time. For example, the opioid overdose reversal medication naloxone may become widely available in control communities prior to the scheduled roll-out time. Therefore premature adoption of a successful intervention will improve the outcome in control communities and attenuate the estimate of the intervention effect if unaccounted for.

## 2. Methods

We first introduce some notation. We denote *Y*_*ij*_ as the summary measure of the outcome for cluster *i* during time period *j*, *i* = 1, …, *N* and *j* = 1, …, *n*, where *N* denotes the number of clusters and *n* denotes the number of time periods in the study, which are assumed to be common to all clusters. In the motivating example, *Y*_*ij*_ is an aggregate count of opioid deaths in county *i* between months *j* and *j* + 1. Using summary measures for the outcome in each cluster has implications when estimating the intervention effect under certain distributional assumptions for the outcome. We discuss this further in Section 2.4.

We assume that the expected outcomes, denoted by *µ*_*ij*_ = *E*[*Y*_*ij*_], come from a generalized linear mixed effects model (GLMM) with link function *g*. In the motivating example we consider *µ*_*ij*_ = *E*[*Y*_*ij*_*/O*_*i*_], where *Y*_*ij*_ assumes a Poisson distribution (*g* = log link), and *O*_*i*_ is an offset for the population size of cluster *i*. We also assume that all clusters receive the full intervention effect immediately after the scheduled implementation, i.e., that the intervention effect does not change with time. We denote the intervention effect by *θ*, and set the corresponding design matrix *X*_*ij*_ = 1 if clusters *i* receives intervention at time *j*, and 0 otherwise.

### 2.1. Random intercept model to adjust for confounding by time

To adjust for confounding by calendar time, Hussey and Hughes recommended incorporation of a time effect in the GLMM:^16^

(1) $$g\left(\mu_{ij}\right)=\alpha +\theta \times X_{ij}+\beta_{j}+b_{0i},$$

where *α* is the intercept, *θ* is the intervention effect, *β*_*j*_ is the discrete time effect, and $b_{0i}\overset{lid}{∼}N\left(0,\sigma_{b0}^{2}\right)$ for *i* = 1, …, *N* are the random intercepts for each cluster.

In some cases, the timing and location of external factors are known in each cluster and their effects can be modeled. Often, investigators are unaware of all factors affecting the outcome. Throughout this paper we assume exposure to these factors are unknown. By incorporating only a single fixed effect for each time step, the standard Hussey and Hughes model requires that the effects of time are common to all clusters, and that the correlation between any two observations in the same cluster are independent of the time step. We note that with proper specification of the time effect, inference based on this model adjusts for such confounding even when it cannot be measured. Therefore, the resulting intervention effect estimate is unbiased. However, correct specification of the random effect structure is necessary for optimal precision.

### 2.2. Random period models to adjust for confounding by time

As misspecification of the time effects can lead to biased estimates of the intervention effect and its standard error, random cluster-by-time period interaction effects have been incorporated in models.^13, 17, 20^ This formulation, referred to as the Hooper/Girling model,^21, 22^ allows the random intercept for each cluster to vary by time period. This model is discussed in Section 2.2.1 below.

#### 2.2.1. Random cluster-by-discrete time effect (uncorrelated, with single variance)

(2) $$g\left(\mu_{ij}\right)=\alpha +\theta \times X_{ij}+\beta_{j}+b_{ij},$$

where $b_{ij}\overset{\text{iid}}{∼}N\left(0,\sigma_{b}^{2}\right)$ are the time-varying random intercepts for each cluster *i* at time period *j*, *i* = 1, …, *N* and *j* = 1, …, *n*. These cluster-specific random effects are intended to capture the effects of confounding factors on the outcome by allowing unique secular trends for each cluster. We note that throughout this paper, we may use the term ”confounding factors” to indicate the presence of external factors that do not differentially impact intervention and control clusters.

There are limitations to the model above that arise from the distributional assumptions regarding the random effects *b*_*ij*_: Constant variance over time, independence of the random effects, and identical correlation between two observations in the same cluster between any two time periods. If exposure to confounding factors increases over time, the variance of the random effect may increase as well. The assumption of independence of the random effects may be violated in the scenario where confounding factors impact clusters at all time intervals after exposure. Such a scenario could lead to greater similarity of random effects during time intervals that are entirely before or entirely after exposure than for random effects for a mix of intervals that are both before and after exposure. Furthermore, while the Hooper/Girling model allows both within-cluster and within-period variation, it imposes a constant correlation across time (for observations in the same cluster).

#### 2.2.2. Random cluster-by-discrete time effect (unstructured covariance)

To account for the limitations described above, we propose an unstructured covariance for the random cluster-by-discrete time interaction terms:

(3) $$g(\mu_{ij})=\alpha +\theta \times X_{ij}+\beta_{j}+b_{ij}^{*},$$

where $b_{i}^{*}=\left(b_{i1}^{*},\ldots ,b_{in}^{*}\right)\overset{\text{iid}}{∼}N\left(0,\Sigma_{b*}\right)$ for *i* = 1, …, *N*. Similar to model 2, the cluster-specific random effects $b_{ij}^{*}$ allow unique secular trends for each cluster. However, the unstructured covariance matrix $\Sigma_{b*}$ imposes no restrictions on the variance across time periods nor on the correlation between the latent time effects within each cluster, albeit with the assumption that the correlation structure is the same for all clusters. While model 3 imposes fewer restrictions than model 2, it requires the estimation of $\frac{(n+1)\times n}{2}$ covariance parameters, which may greatly reduce the power to detect an intervention effect and may lead to issues of identifiability of regression parameter estimates.

#### 2.2.3. Random cluster by linear time effect

An alternative to models 2 and 3 is to include a random slope for time in each cluster:

(4) $$g\left(\mu_{ij}\right)=\alpha +\theta \times X_{ij}+\beta_{j}+b_{0i}+b_{1i}\times t_{j},$$

where $\left(b_{0i},b_{1i}\right)∼N\left(0,\Sigma_{b_{0},b_{1}}\right)$ and $\Sigma_{b_{0},b_{1}}$ is the 2 × 2 covariance matrix for (*b*_0*i*_, *b*_1*i*_), *i* = 1, *…, N*, and *t*_*j*_ is the time between the study start date and the beginning of time period *j*. The random effects *b*_1*i*_ allow for unique linear time trends in each cluster. Model 4 assumes the variance in the outcome changes monotonically with time and imposes restrictions on the correlation between observations within a cluster, and is therefore more restrictive than model 3.

### 2.3. Random group-by-period models to adjust for confounding by time and early adoption or time-varying effect modification

The models described in Section 2.2 are insufficient for the setting where external factors differentially impact intervention and control clusters. This scenario may arise when such events lead to premature adoption of intervention components by control clusters - a form of intervention noncompliance. We refer to this as early adoption, and assume this is unbeknownst to the investigator. To model this scenario, we need to include group×time interaction terms, i.e., different fixed and random effects for control and intervention clusters. Unlike group×time interaction models for time-varying treatments, these models accommodate situations in which secular trends systematically differ between intervention and control clusters.

In models 5 through 7, we incorporate a fixed discrete time effect, *β*_*j*_, that is common to all clusters. Because incorporating a group-by-discrete time interaction may lead to loss of power and potentially also to nonidentifiability, we include a fixed linear time effect in the control group by incorporating the term $\gamma \times t_{j}\times 1_{\left\{X_{ij}=0\right\}}$ in models 5 through 7. In this setting, incorporation of a linear time effect in the control group models the effect of early adoption of a beneficial intervention on overdose death rates as a monotonic decrease with time.

We note that these same set of models would also be appropriate for the setting of time-varying effect modification. An example would be changes in insurance policy that either enhance the effect of the intervention (because access to opioid treatments that are not part of the intervention is reduced in control clusters) or reduce it (because such access is increased in control clusters). Both scenarios can be accommodated by the models below. In these models, the estimate of *θ* provides the intervention effect estimate, and *γ* captures the effect of early adoption. When time-varying effect modification is present, the parameter *γ* captures the difference in the effect of external factors between intervention and control groups. We discuss these models further in Sections 2.3.1 through 2.3.3.

#### 2.3.1. Random cluster-by-intervention-by-discrete time effect (uncorrelated, with single variance for each group)

(5) $$g\left(\mu_{ij}\right)=\alpha +\theta \times X_{ij}+\beta_{j}+\gamma \times t_{j}\times 1_{\left\{X_{ij}=0\right\}}+b_{ij}+c_{ij}\times 1_{\left\{X_{ij}=0\right\}},$$

where *γ* × *t*_*j*_ is the difference in the time effect between control and intervention clusters at time period *j*, and $c_{ij}∼N\left(0,\sigma_{c}^{2}\right)$ is a random time effect for control cluster *i* at time period *j* for *i* = 1, …, *N* and $j=1,\ldots ,t_{i}^{I}$, where $t_{i}^{I}$ is the time period in which the intervention was scheduled to be rolled-out to cluster *i*. For control clusters that prematurely adopt intervention components, the cluster-specific random effects (*c*_*ij*_) would be expected to be larger (in magnitude) than those that do not. Model 5 is an extension to Model 2 (Hooper/Girling), in which the variance of the cluster-by-time period random effects systematically differ between intervention and control clusters. This model is subject similar limitations as those discussed in Section 2.2.1.

#### 2.3.2. Random cluster by-intervention-by-discrete time effect (unstructured covariance for each group)

To account for the limitations of model 5, we propose allowing an unstructured covariance for the random cluster-by-discrete time interaction terms in each group:

(6) $$g\left(\mu_{ij}\right)=\alpha +\theta \times X_{ij}+\beta_{j}+\gamma \times t_{j}\times 1_{\left\{X_{ij}=0\right\}}+b_{ij}^{*}+c_{ij}^{*}\times 1_{\left\{X_{ij}=0\right\}},$$

where $c_{i}^{*}=\left(c_{i1}^{*},\ldots ,c_{i,t_{i}^{I}}^{*}\right)\overset{\text{iid}}{∼}N\left(0,\Sigma_{c_{i}^{*}}\right)$ for *i* = 1, …, *N*. Here $\Sigma_{c_{i}^{*}}$ consists of the first $t_{i}^{I}$ rows and columns of $\Sigma_{c^{*}}$, where $\Sigma_{c^{*}}$ is the unstructured covariance matrix for a control cluster scheduled to receive the intervention during the final period of the roll-out phase. Similar to model (5), the cluster-specific random effects, $c_{ij}^{*}$, are intended to capture the effects of premature exposure to intervention components. This is an extension of model 3, which assumes the random cluster-by-discrete time effects shared a common (unstructured) covariance matrix. While the random effect covariance structure in model 6 has less restrictions than in model 5, it requires the estimation of $\frac{\left(n+1)\times n+(n_{\tau }-1)\times (n_{\tau }-2\right)}{2}$ covariance parameters and is subject to similar limitations as discussed in Section 2.2.2. Here we define *n*_*τ*_ as the number of time periods prior to the follow-up phase of the study.

#### 2.3.3. Random cluster by-intervention-by-linear time effect

Similar to model 4, we can impose a random slope for time in each group.

(7) $$g\left(\mu_{ij}\right)=\alpha +\theta \times X_{ij}+\beta_{j}+\gamma \times t_{j}\times 1_{\left\{X_{ij}=0\right\}}+b_{0i}+b_{1i}\times t_{j}+\left(c_{0i}+c_{1i}\times t_{j}\right)\times 1_{\left\{X_{ij}=0\right\}},$$

where $\left(c_{0i},c_{1i}\right)∼N\left(0,\Sigma_{c_{0},c_{1}}\right)$ and $\sum_{c_{0},c_{1}}$ is the 2 × 2 covariance matrix for (*c*_0*i*_, *c*_1*i*_), *i* = 1, …, *N*. The cluster-specific random effects *c*_1*i*_ allow for unique linear time trends in each control cluster and are intended to capture the effects of premature adoption of intervention components.

#### 2.3.4. Fixed effects for linear time in all clusters

To further limit loss of power and potential nonidentifiability due to the large number of parameters, we replace the fixed discrete time effect in models 5 through 7, *β*_*j*_, with a fixed linear time effect, *β* × *t*_*j*_, in models 8 through 10. This strategy may be useful in situations when the combined effects of external factors result in monotonic changes in the outcome over time (e.g., rising tide). For example, the implementation of a new policy intended to reduce opioid prescriptions in conjunction with media awareness campaigns may decrease the level of opioid overdose deaths with time.

(8) $$g\left(\mu_{ij}\right)=\alpha +\theta \times X_{ij}+\beta \times t_{j}+\gamma \times t_{j}\times 1_{\left\{X_{ij}=0\right\}}+b_{ij}+c_{ij}\times 1_{\left\{X_{ij}=0\right\}}$$

(9) $$g(\mu_{ij})=\alpha +\theta \times X_{ij}+\beta \times t_{j}+\gamma \times t_{j}\times 1_{\left\{X_{ij}=0\right\}}+b_{ij}^{*}+c_{ij}^{*}\times 1_{\left\{X_{ij}=0\right\}}$$

(10) $$g\left(\mu_{ij}\right)=\alpha +\theta \times X_{ij}+\beta \times t_{j}+\gamma \times t_{j}\times 1_{\left\{X_{ij}=0\right\}}+b_{0i}+b_{1i}\times t_{j}+\left(c_{0i}+c_{1i}\times t_{j}\right)\times 1_{\left\{X_{ij}=0\right\}}$$

### 2.4. Estimation

The estimands of interest in our setting may depend on the goals of the study and the nature of the time-varying external factors that impact the study. In the setting where only confounding by time is of concern, the causal estimand is the average (across clusters) intervention effect on the outcome of interest– which is assumed to be fixed. Unbiased estimation is made possible by inclusion of a fixed time effect as described above. In the setting of early adoption, the causal estimand of interest to investigators might also be the average intervention effect (compared to the control condition with no early adoption), but unbiased estimation is only possible if either we can accurately model the effect as a function of time or can measure the amount of early adoption (we return to this point in the discussion). Alternatively, investigators might be interested in the randomized intervention effect as a function of time, which would likely wane with time because of the early adoption. Finally we consider the setting where time-varying external factors are effect modifiers. Here investigators might, once again, be interested in a causal estimand that is the average effect of intervention as a function of time. As before, the effect of the external factors must be modeled in order to obtain unbiased estimates of this estimand. We note that although spline and other flexible models might be used, we considered only the parametric models described above for inference. To investigate their robustness, we use different models for data generation (the mechanism of which is generally unknown) than for inference.

The models for inference in Section 2 can be fit by specifying the Poisson family in the *glmer* function (package: *lme4*) in ***R***.^25^ Of note, when the outcome is a summary measure for each cluster (as is the case in our motivating example), we ran into estimation difficulties when specifying random cluster-by-discrete time effects when the outcome distribution was assumed to be Gaussian. This is due to incorporation of a residual term for each cluster for each time period, which yields unidentifiable random effects. This issue does not arise under Poisson distributional assumptions, since the variance is directly proportional to the mean and thus residual terms are not estimated. To accommodate the Gaussian distribution assumption in this scenario, multiple communities per cluster^13, 17, 20^ or multiple intervals per time period would be needed.

In the models above, the fixed time effects *β*_*j*_ and *β* are modeled to capture confounding by time. The fixed time effects *γ* captures the effect of early adoption or time-varying effect modification. These effects are all identifiable because the design matrices are full rank. Without additional assumptions, however, the above models cannot distinguish among the effects of external factors, early intervention adoption, or of other time-varying factors. Rather, they model temporal trends that may be a consequence of these factors; such models allow for assessment of the impact of such trends on bias, power, and Type 1 error. When used in analysis, our simulation studies demonstrate that these models can reduce bias in estimation of intervention effects, in the settings appropriate for their use.

## 3. Simulation study

We conduct a simulation study to investigate the impact of external factors and noncompliance on the bias, coverage probability of 95% confidence intervals, power, and Type 1 error of the intervention effect estimate. The data are simulated based on the motivating example described in Section 1.1. In this setting, we have *N* = 18 clusters and *n* = 13 time periods. The intervention is rolled out to each cluster according to the time line provided in Figure 1. In all data generating models, the outcomes *Y*_*ij*_ are simulated from a Poisson distribution, and represent the number of opioid overdose deaths in cluster *i* during time period *j*. Here *g* is the log link, and the population size of cluster *i*, *O*_*i*_, is included as an offset in all models. That is, *g*(*µ*_*ij*_) = *log*(*E*[*Y*_*ij*_]*/O*_*i*_). The intercept *α* is set to −10 and the standard deviation of the random intercept, $\sigma_{b_{0}}$, is set to 0.30. These numbers were determined using the opioid death counts in the 18 South Carolina clusters between 2016 and 2018. The data are provided in Supplementary Table 1. The intervention effect *θ* is set to log(0.6), which represents the target 40% reduction in opioid overdose deaths due to intervention (Section 1.1).

### 3.1. Simulation scenarios

We apply models 1 through 10 under four general scenarios: (1) standard, (2) confounding, (3) early adoption, and (4) confounding + early adoption or effect modification. We set the number of simulations for each scenario at 500. The data generation process for each scenario is summarized in Table 1, and is described in more detail below. It is important to distinguish between the data generation models described in this section, which are used to simulate the data, and the analysis models (for inference) described in Section 2, which are used to analyze the data. In these simulations, we intentionally generate the data from different models than those used for analysis. For example, we simulate the effect of confounding due to a rising tide by randomly selecting communities exposed to the underlying external event at each time point, and for each community, randomly generating the impact of the confounding factors on outcomes at each time point. This allows for investigation of the robustness of the proposed analysis models to both the parameterization of the fixed and random effects and the underlying processes (e.g., external factors, early adoption, etc.) that cause confounding, noncompliance, and/or effect modification.

In the first scenario (standard) we assume no confounding, early adoption, or effect modification is present; all clusters receive the intervention during the scheduled time period and no external factors influence the outcome. The data are generated according to scenario 1 in Table 1. The data generation model for scenario 1 is the same as (analysis) model 1 defined in Section 2.1, with *β*_*j*_ = 0 for *j* = 1, …, *n*.

In the second scenario, all clusters not currently exposed to confounding factors by time period *j* have a 1 in N chance of exposure. Once exposed, each cluster continues to be exposed through the remainder of the study period. This is intended to reflect the situation where confounding factors have long-lasting effects, such as new public policies limiting opioid prescriptions and media awareness campaigns. The number of clusters exposed to confounding factors at each time period *j*, ${n^{\prime }}_{j}$, is simulated from a *Binomial $\left({N^{\prime }}_{j},1/N\right)$* distribution, where ${N^{\prime }}_{j}$ is the total number of clusters unexposed to the confounding factors prior to time period *j*.

Once exposed, the effect of confounding factors on the outcome in cluster *i* is set to vary uniformly at each time period *j*. Specifically, we let ${t^{\prime }}_{i}$ denote the time period in which cluster *i* is exposed to confounding factors. We simulate *β*_*ij*_ under 2 settings. For $j\geq {t^{\prime }}_{i}$, we simulate *β*_*ij*_ ∼ *unif* [−1, 0], corresponding to a positive effect of confounding factors on the outcome opioid overdose deaths (scenario 2.1). This is intended to represent a rising tide of events aimed at improving outcomes. We set *β*_*ij*_ = 0 for $j<{t^{\prime }}_{i}$, indicating that cluster *i* has yet to be exposed to confounding factors. To represent a negative impact of confounding factors on the outcome, we simulate *β*_*ij*_ ∼ *unif* [0, 1] for $j\geq {t^{\prime }}_{i}$ and *β*_*ij*_ = 0 for $j<{t^{\prime }}_{i}$ (scenario 2.2).

In the third scenario (early adoption), the number of clusters prematurely adopting intervention components during each time period *j*, $n_{j}^{*}$, is simulated from a *Binomial* $\left(N_{j}^{*},\frac{N-N_{j}^{*}+1}{2\times N}\right)$ distribution. Here $N_{j}^{*}$ is the number of control clusters which have not adopted any intervention components by time period *j*. This set up reflects the nature of SWD, where the number of control clusters at risk for early adoption decreases with time as more clusters crossover to the intervention group by design. This formulation allows the probability of exposure to intervention components increase at each time point *j* (for an unexposed cluster). This situation is feasible in certain settings, such as when intervention components become more widely available with time, thus increasing the probability of exposure for a control cluster.

For all time periods *j* in which a cluster is subjected to early adoption, the magnitude of the early intervention adoption effect is set to vary uniformly. Denote $t_{i}^{*}$ as the time period of early adoption for cluster *i* and denote $t_{i}^{I}$ as the time period in which the intervention was scheduled to be rolled-out to cluster *i*. We simulate *θ*_*ij*_ ∼ *unif* [*θ*, 0] for $j=t_{i}^{*},\ldots ,t_{i}^{I}-1$, and *θ*_*ij*_ = 0 otherwise. Thus the maximum effect of early intervention adoption on the outcome for exposed control clusters is set to *θ*. In this scenario, the event (i.e., early adoption) has a positive effect on the outcome in the control group only.

In the fourth scenario, the effects of confounding factors and early adoption are simulated in the same manner as in scenario 2 and scenario 3, respectively. In scenario 4.1, confounding factors have a positive effect on the outcome. In scenario 4.2, confounding factors have a negative effect on the outcome. The negative impact of confounding factors on the outcome is partially offset by the positive impact of early adoption on the outcome (for control communities) in scenario 4.2. The data generation model for time-varying effect modification is similar to the generation model for simultaneous confounding and early adoption described in scenario 4. Here, the parameters *β*_*ij*_ would be interpreted as the effects of external factors on the outcomes in the intervention communities, and *θ*_*ij*_ would be interpreted as the differences in the effects of external factors between intervention and control communities.

### 3.2. Results

Simulation results are presented in Table 2. Under scenario 1 (standard), all models yield unbiased estimates of the intervention effect, reach nominal confidence interval coverage rates, and preserve Type 1 error. Model 1, which does not include any random effects for time, yields unbiased estimates of the intervention effect in scenario 2 (confounding), where external factors do not differentially impact control and intervention clusters. However, coverage probabilities are below the nominal level of 0.95 and Type 1 error is inflated. Model 1 is heavily biased in scenarios 3 (early adoption) and 4 (confounding & early adoption), where external factors differentially impact intervention and control clusters.

Models 2 through 4, which include random cluster-by-time effects, yield unbiased estimates of the intervention effect when external factors do not differentially impact control and intervention clusters (i.e., scenarios 1 and 2). Model 3, which assumes an unstructured covariance for the random cluster-by-time interactions, performs the best with regard to coverage probabilities of the 95% confidence intervals and Type 1 error preservation. Model 2, which assume a single variance for the random cluster-by-time interactions (Hooper/Girling model), perform slightly worse on these metrics. Model 4, which assume a random slope for time for each cluster, has the highest inflation in Type 1 error with coverage probabilities well below the nominal level of 0.95. Models 2 through 4 are overpowered for scenarios 1 and 2. These models perform poorly when early adoption is present in scenarios 3 and 4 (i.e., when external factors differentially impact intervention and control clusters). The estimated intervention effect is reduced by 31.8% to 33.6% in these scenarios.

**Figure 2.**
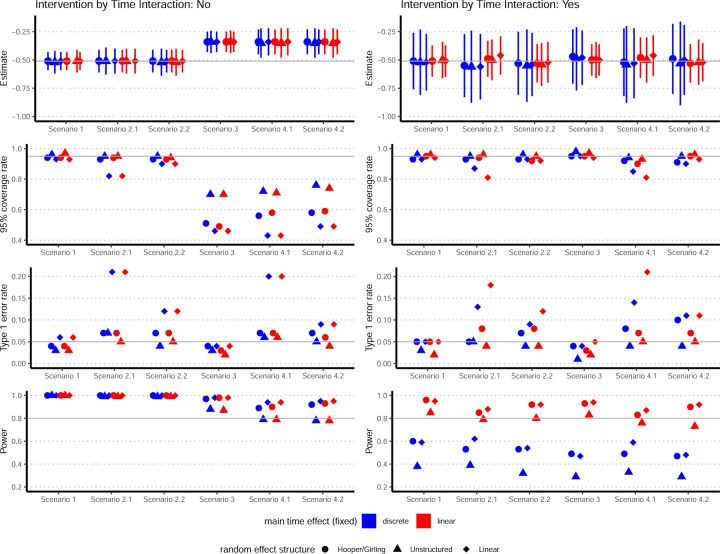
Performance of models with and without fixed and random intervention-by-time interactions. Models are compared across scenarios listed in Table 1. First row compares intervention effect estimates ± empirical standard error. Horizontal gray line: true intervention effect *θ* = log(0.6). Second, third, and fourth rows compare models on coverage rate of 95% confidence intervals, Type 1 error rate, and power, respectively. Horizontal gray lines indicate 95% coverage rate, 0.05 Type 1 error rate, and a power of 0.80 in the second, third, and fourth rows, respectively. Covariance structure for cluster-by-time random effects: Hooper/Girling labeled by circles, unstructured labeled by triangles, and linear labeled by diamonds. Models which incorporate a discrete term for the main effect for time (fixed) are labeled by blue shapes; models which incorporate a linear term are labeled by red shapes. Models without intervention-by-time interactions displayed in left column, where blue shapes correspond to models 2 through 4 in Section 2. Models with intervention-by-time interactions displayed in right column, and correspond to models 5 through 10 in Section 2. Models 5 through 10 include a linear time effect for the fixed intervention-by-time interaction term.

The performance of models with and without intervention-by-time interactions is compared in Figure 2. Models without intervention-by-time interactions are displayed in the left column, where the blue shapes correspond to models 2 through 4. For comparison, we include models which replace the discrete main effect for (fixed) time in models 2 through 4, *β*_*j*_, with the linear fixed effect *β* × *t*_*j*_. These models are labeled by the red shapes in the left column of Figure 2. The models in the right column of Figure 2 include fixed and random intervention-by-time interactions. These models correspond to models 5 through 10.

Models 5 through 10 perform well in all simulation scenarios. When no confounding or early adoption is present (scenario 1), these models yield little bias, reach nominal coverage probabilities for 95% confidence intervals, and preserve Type 1 error. When only confounding factors are present (scenario 2), all models have less than 10% bias. These models generally reach nominal confidence interval coverage probabilities and preserve Type 1 error. The exceptions are models 7 and 10, which include random cluster-by-group-by-linear time interaction effects.

When external factors differentially impact intervention and control clusters due to early adoption (scenarios 3 and 4), models 5 through 10 greatly reduce the bias in the intervention effect estimate compared to models which do not include intervention-by-time interactions. Models 6 and 9, which assume an unstructured covariance for the random cluster-by-group-by-discrete time interactions, generally yield the lowest bias, while reaching nominal coverage probabilities of the 95% confidence intervals and preserving Type 1 error. Models 5 and 8 (Hooper/Girling models) perform slightly worse on these metrics. Models 7 and 10 have the highest inflation in Type 1 error and coverage probabilities well below the nominal level.

The choice of a discrete or linear term for the fixed time effect does not impact power in models without intervention-by-time interactions. This result is expected given the findings of Grantham et al.^26^ For models which include intervention-by-time interaction terms (i.e., models 5 through 10), the strongest correlate of power is the fixed effect for time. Models with discrete time effects, which incorporate a parameter for each time period, have much lower power compared to models which incorporate a single parameter corresponding to a linear time effect. For a given fixed time effect, models with a random slope for time achieve the highest power (model 7 among fixed discrete time effects; model 10 among fixed linear time effects). Models which assume an unstructured covariance for random cluster-by-time effects achieve the lowest power (model 6 among fixed discrete time effects; model 9 among fixed linear time effects).

## 4. Discussion

SWDs and alternative cluster randomized trials are currently being used to implement interventions to reduce opioid overdose deaths in communities across several states. However, these interventions are competing with newly initiated public policies and media awareness campaigns. Furthermore, control communities may adopt components of proposed interventions as they become readily available. These scenarios induce confounding by time, treatment noncompliance, and time-varying effect modification. Given the difficulties in capturing the timing and exposure levels of external factors, we considered mixed-effects models which incorporate fixed and random time effects to account for these latent factors. We discussed the limitations of commonly used models in the context of proposed SWDs to combat the opioid epidemic, and proposed solutions to accommodate deviations from these assumptions.

Through our simulation study, we showed that mixed effects models are sensitive to the scenarios considered here (i.e., confounding, noncompliance, and effect modification). While the Hooper/Girling model^17, 20^ offers an improvement over the standard Hussey and Hughes model,^16^ it may result in severely biased estimates of the intervention effect when secular trends systematically differ between intervention and control clusters. Even in scenarios where these models do capture the secular trend, we demonstrated how incorrect specification of the cluster-level covariance over time can yield under coverage of confidence intervals and inflation of Type 1 error. Similar conclusions have been reached in other studies.^3, 22, 22, 27, 28^

Alternatively, models which allow secular trends to systematically differ between the intervention and control clusters through incorporation of fixed and random group-by-time effects offer a major improvement in terms of bias reduction. Our simulation studies confirmed that models incorporating unstructured cluster-level covariances for the random intervention-by-cluster-by-time interaction terms yielded nominal confidence interval coverage rates and preserved Type 1 error (i.e., models 6 and 9). However, these models may be underpowered for certain parameterizations of the fixed time effects.

The proposed mixed-effects modeling framework discussed in this paper treats external factors as latent processes; these are accounted for through the incorporation of fixed and random time effects. This modeling strategy is useful in practice because investigators are often unaware of all external factors affecting the outcome, let alone each cluster’s level of exposure to these factors. For the models considered in this study, correct specification of the fixed time effects were primarily responsible for the attenuation of bias in the intervention effect estimate.

Our results also have potential implications for data collection in stepped-wedge studies. As previously noted, unbiased estimation of the estimands of interest in settings of time-varying effect modification and early adoption rely on assumptions about these effects. Information collected during the study on the processes of interest could also be incorporated in models. Doing so is more straightforward in a setting in which external events, such as changes in insurance policy, cannot be affected by outcomes on the study. For issues such as early adoption of treatment, one must be concerned about the potential “confounding by indication” wherein participants or their providers might make choices based on the individual characteristics as they vary over time. In this context, unbiased estimation of the average intervention effect would require correct modeling of the selection mechanism as a function of measured confounders for early intervention adoption. An alternative estimand, which is simply the randomized intervention effect as a linear function of time (e.g., models 8–10) might also be of interest; interpretation of such results might also benefit from knowledge of the extent of early adoption in the population of interest.

### 4.1. Limitations and Future Research

An important limitation of the models we discussed in this paper is that they assume the intervention effect does not vary with time. Models attempting to account for both secular trends which differ by intervention group, and time-varying treatment effects, may lead to unidentifiable intervention effect estimates. Furthermore, the approach discussed here is limited to GLMM. Generalized estimating equations (GEE) also allow for correlation in the outcomes, and are robust to misspecification of the covariance structure.^29^ Ren et al. show that GEE is more robust to model misspecification than linear mixed models when the random intercepts differ by intervention group. Future work is needed to explore the performance of GEE models in the context considered here, where secular trends in the control and intervention clusters arise from different mechanisms, and differentially impact clusters within each group. Several nonparametric methods have also been proposed that use within-period^3, 27, 30^ or between period^3^ comparisons to account for confounding by time. While these models are robust to misspecification of the random effects, the performance of these models when external factors differentially impact intervention and control clusters, such as in the case of early intervention adoption, has not been explored.

Although our paper focused on stepped-wedge designs, the secular trends in outcomes induced by rising tides and early intervention adoption may also be present in other types of cluster randomized trials. Simulation studies are needed to determine the impact of such scenarios on the bias of the intervention effect estimate, Type 1 error, and power in these settings. We note that Grantham et al. establish a sufficient condition for when the choice of time parameterization does not impact the variance of the estimated intervention effect in cluster randomized trials.^26^ This information can be useful in the planning of such trials. While Grantham et al establish that categorical or linear fixed time effects do not impact the variance estimator of the intervention effect in SWD, this was not the case when group-by-time interaction terms were modeled (as demonstrated by our simulation study). Future investigation is needed to establish sufficient conditions in the presence of interactions.

### 4.2. Conclusion and Recommendations

Stepped-wedge designs are particularly suitable for epidemics and pandemics, where complex health interventions are needed as soon as possible. Given the urgency of such situations, other policies and interventions aimed at improving outcomes will likely be implemented in concurrence with any proposed interventions. Studies must therefore consider these issues during the planning stages. We have shown that models which incorporate fixed and random group-by-time effects are effective in reducing bias when external factors differentially impact intervention and control clusters. However, incorporation of such time effects may impact power and must be accounted for in sample size calculations. Models incorporating an unstructured covariance for the random intervention-by-cluster-by-time interaction effects are most effective in reducing bias, reaching nominal confidence interval coverage, and preserving Type 1 error; but they can lead to severely under powered studies when used in conjunction with discrete fixed time effects. One strategy is to use fixed parametric intervention-by-time effects with the unstructured covariance; these models perform reasonably well in the scenarios considered here. Alternatively, one can impose a more restrictive covariance structure such as exponential decay over time, Hooper/Girling covariance, or random slopes for time. To account for potential under coverage of confidence intervals and Type 1 error inflation, randomization based inference should be used.^31, 32^ Simulation studies may be needed to estimate sample size and power for certain models, as formulas to calculate them are not currently available for many random effect covariance structures.

## Supplementary Material

Supplementary Material

## Figures and Tables

**Table 1.** Data generation models for simulations under each scenario.

Scenario	Data generating model and scenario description	Impact on outcome	Index
Standard	log(*µ*_*ij*_) = *α* + *θ* × *X*_*ij*_ + *b*_*i*_ **Description**: No confounding, early adoption, or effect modification	None.	1
Confounding	log(*µ*_*ij*_) = *α* + *θ* × *X*_*ij*_ + *β*_*ij*_ + *b*_*i*_ **Description**: At each time period *j*, ${n^{\prime }}_{j}$ clusters randomly exposed to event inducing confounding for remainder of study period; ${n^{\prime }}_{j}∼Binomial\left({N^{\prime }}_{j},1/N\right)$, where ${N^{\prime }}_{j}$ is total number of clusters unexposed to event prior to time period *j*.	*β*_*ij*_ ∼ *unif* [−1, 0] if cluster *i* exposed during time period *j*; 0 otherwise.	2.1
		*β*_*ij*_ ∼ *unif* [0, 1] if cluster *i* exposed during time period *j*; 0 otherwise.	2.2
Early adoption	*$\text{log}\left(\mu_{ij}\right)=\alpha +\theta_{ij}\times 1_{\left\{X_{ij}=0\right\}}+\theta \times X_{ij}+b_{i}$* **Description**: At each time period *j*, $n_{j}^{*}$ control clusters prematurely adopt intervention components; $n_{j}^{*}∼Binomial\left(N_{j}^{*},\frac{N-N_{j}^{*}+1}{2\times N}\right)$, where $N_{j}^{*}$ is the number of control clusters not receiving the intervention prior to time period *j*.	*θ*_*ij*_ ∼ *unif* [*θ*, 0] if control cluster *i* prematurely adopts intervention at time period *j*; 0 otherwise.	3
Confounding + Early adoption (or Effect modification)	*$\text{log}\left(\mu_{ij}\right)=\alpha +\beta_{ij}+\theta_{ij}\times 1_{\left\{X_{ij}=0\right\}}+\theta \times X_{ij}+b_{i}$* **Description**: At each time period *j*, ${n^{\prime }}_{j}$ clusters are randomly exposed to confounding events and $n_{j}^{*}$ control clusters prematurely adopt intervention components, where $n^{\prime }$ and $n_{j}^{*}$ are defined above. Control clusters may be exposed to both confounding factors and early adoption. Data generation model for effect modification is similar.	*β*_*ij*_ ∼ *unif* [−1, 0] if cluster *i* exposed to confounding event during time period *j*; 0 otherwise. *θ*_*ij*_ is defined as above.	4.1
		*β*_*ij*_ ∼ *unif* [0, 1] if cluster *i* exposed to confounding event during time period *j*; 0 otherwise. *θ*_*ij*_ is defined as above.	4.2

Data is simulated under 4 general scenarios. The data generating model for each simulation scenario is displayed in the second column. Here *µ*_*ij*_ is the expected rate of opioid overdose deaths in cluster *i* during time period *j*, *θ* is the intervention effect and is set to log(0.6), and *X*_*ij*_ is an indicator of whether cluster *i* is scheduled to receive intervention during time period *j* and is based on the SWD represented by Figure 1. The fixed intercept *α* is set to −10 and the random intercept *b*_*i*_ is simulated from a *N* (0, 0.30) distribution. A description of the selection process for exposure to confounding events or early adoption is provided in the second column (below the data generating model). The impact of confounding factors and/or early adoption on the outcome is detailed in the third column. In scenarios 2 and 4, we allow confounding factors to have either a positive impact on the outcome (scenarios 2.1 and 4.1) or a negative impact on the outcome (scenarios 2.2 and 4.2).

**Table 2.** Simulation Results.

Model index	Time effects				Scenario 1						Scenario 2.1						Scenario 2.2						

	Main		Interaction		Standard						Confounding (+)						Confounding (−)						

	fixed	random	fixed	random	%bias	SD	SE	cov	pwr	*α*	%bias	SD	SE	cov	pwr	*α*	%bias	SD	SE	cov	pwr	*α*	
1	disc				0.4	0.08	0.08	0.94	1	0.05	−0.5	0.11	0.08	0.83	1	0.20	0.7	0.10	0.09	0.92	1	0.10	%
2	disc	H/G			0.3	0.08	0.08	0.94	1	0.04	−0.4	0.10	0.10	0.93	1	0.07	0.7	0.10	0.09	0.93	1	0.07	
3	disc	UN			0.9	0.10	0.11	0.96	1	0.03	0.6	0.12	0.12	0.95	0.99	0.07	2.3	0.12	0.12	0.95	0.99	0.04	
4	disc	lin			0.5	0.08	0.08	0.93	1	0.06	0.1	0.11	0.08	0.82	1	0.21	0.5	0.10	0.09	0.90	1	0.12	
5	disc	H/G	lin	H/G	0.6	0.25	0.24	0.93	0.6	0.05	8.0	0.28	0.26	0.93	0.53	0.05	4.2	0.28	0.26	0.93	0.53	0.07	
6	disc	UN	lin	UN	1.5	0.29	0.31	0.96	0.38	0.03	9.3	0.32	0.33	0.95	0.39	0.05	6.8	0.32	0.35	0.96	0.32	0.04	
7	disc	lin	lin	lin	0.9	0.25	0.24	0.93	0.59	0.05	9.2	0.29	0.23	0.87	0.62	0.13	5.0	0.28	0.26	0.93	0.54	0.09	
8	lin	H/G	lin	H/G	−0.4	0.14	0.14	0.95	0.96	0.05	−4.7	0.16	0.16	0.94	0.85	0.08	4.5	0.17	0.16	0.92	0.92	0.08	
9	lin	UN	lin	UN	−1.5	0.16	0.17	0.96	0.85	0.02	−2.7	0.18	0.19	0.96	0.79	0.04	5.3	0.19	0.2	0.95	0.8	0.04	
10	lin	lin	lin	lin	−0.8	0.14	0.14	0.94	0.95	0.05	−9.8	0.17	0.13	0.81	0.88	0.18	2.2	0.18	0.15	0.92	0.92	0.12	
Model index	Time effects				Scenario 3						Scenario 4.1						Scenario 4.2						

	Main		Interaction		Early adoption						Confounding (+) & Early adoption						Confounding (−) & Early adoption						

	fixed	random	fixed	random	%bias	SD	SE	cov	pwr	*α*	%bias	SD	SE	cov	pwr	*α*	%bias	SD	SE	cov	pwr	*α*	

1	disc				−33.6	0.09	0.09	0.49	0.98	0.04	−33.1	0.12	0.08	0.44	0.94	0.20	−32.8	0.11	0.09	0.53	0.95	0.10	
2	disc	H/G			−33.6	0.09	0.09	0.51	0.97	0.04	−33.4	0.11	0.10	0.56	0.89	0.07	−32.8	0.11	0.10	0.58	0.92	0.07	
3	disc	UN			−32.7	0.10	0.11	0.7	0.88	0.03	−32.3	0.13	0.12	0.72	0.79	0.06	−31.8	0.13	0.13	0.76	0.78	0.05	
4	disc	lin			−33.4	0.09	0.08	0.46	0.98	0.04	−33.0	0.12	0.08	0.43	0.94	0.20	−32.8	0.11	0.09	0.49	0.95	0.09	
5	disc	H/G	lin	H/G	−8.0	0.24	0.24	0.95	0.49	0.04	2.1	0.30	0.27	0.92	0.49	0.08	−4.0	0.31	0.27	0.91	0.47	0.10	
6	disc	UN	lin	UN	−3.9	0.28	0.32	0.98	0.29	0.01	4.8	0.34	0.34	0.94	0.33	0.04	2.9	0.37	0.37	0.95	0.29	0.04	
7	disc	lin	lin	lin	−6.8	0.24	0.24	0.95	0.47	0.04	4.2	0.31	0.24	0.85	0.59	0.14	−2.4	0.31	0.26	0.9	0.48	0.11	
8	lin	H/G	lin	H/G	−3.0	0.14	0.14	0.95	0.93	0.03	−6.6	0.18	0.16	0.90	0.83	0.07	2.9	0.17	0.16	0.95	0.90	0.07	
9	lin	UN	lin	UN	−1.9	0.16	0.18	0.97	0.83	0.02	−1.8	0.20	0.19	0.93	0.76	0.05	2.5	0.20	0.20	0.96	0.73	0.05	
10	lin	lin	lin	lin	−2.9	0.14	0.14	0.94	0.94	0.05	−10.8	0.18	0.13	0.81	0.87	0.21	1.0	0.17	0.15	0.93	0.92	0.11	

bias $\left(\frac{\hat{\theta }-\theta }{\theta }\right)$, standard deviation (SD), estimated standard error (SE), coverage rate of 95% confidence interval (cov), power (pwr), and Type 1 error (*α*) of estimated intervention effect. Rows correspond to fitting models 1 through 10 under the scenarios described in Table 1; +/− imply positive/negative effect of confounding factors on outcome. Models include discrete (disc) or linear (lin) fixed time effects for all clusters (main effect) and/or control clusters (interaction effect). H/G, UN, and lin indicate Hooper/Girling, unstructured, and linear random effect structure for time effects.
