# Supplementary material for: Accounting for confounding by time, early intervention adoption, and time-varying effect modification in the design and analysis of stepped-wedge designs: Application to a proposed study design to reduce opioid-related mortality
Source: Res Sq. 2020 Nov 12:rs.3.rs-103992. Preprint. [Version 1] doi: 10.21203/rs.3.rs-103992/v1 (PMC7668751; doi:10.21203/rs.3.rs-103992/v1)
Supplement: Supplementary Material [file 5120d2e3737613967e83ba65.pdf]

Supplementary Table 1: Opioid overdose in the 18 highest risk communities in South Carolina betv

	Opioid overdose deaths			
Community	2016	2017	2018	Population
Aiken	23	31	27	169401
Anderson	20	22	15	200482
Beaufort	8	18	14	188715
Berkeley	26	23	24	221091
Charleston	65	94	100	405905
Dorchester	14	20	30	160647
Florence	15	26	26	138159
Georgetown	27	14	16	62249
Greenville	53	73	131	514213
Greenwood	7	14	19	70741
Horry	101	77	85	344147
Lancaster	5	23	12	95380
Lexington	33	36	42	295032
Oconee	10	18	15	78374
Pickens	25	19	22	124937
Richland	42	71	51	414576
Spartanburg	36	47	56	313888
York	42	45	43	274118

veen 2016 and 2018
